# Urolithin A-Enhanced Multi-Bioactive Formulation Mitigates Cyclophosphamide-Induced Premature Ovarian Failure Through Suppression of Oxidative-Inflammatory Stress and Preservation of Follicle Fate

**DOI:** 10.3390/antiox15060662

**Published:** 2026-05-24

**Authors:** Yangyan Dai, Silu Zhang, Lijia Yang, Penglong Liu, Tingfeng Zhang, Hailong Li, Yuchen Pang, Shijing Ma, Yehui Zhang, Tiantian Zhao

**Affiliations:** 1 WONDERLAB Institute of Microecology and Nutrition, Shenzhen Porshealth Bioengineering Co., Ltd., Shenzhen 518000, China; yangyan.dai@wonderlab.top (Y.D.); echo.zhang@wonderlab.top (S.Z.); hailong.li@wonderlab.top (H.L.); janis.pang@wonderlab.top (Y.P.); mashijing@wonderlab.top (S.M.); 2 Sericulture & Agri-food Research Institute Guangdong Academy of Agricultural Sciences, Key Laboratory of Functional Foods, Ministry of Agriculture and Rural Affairs, Guangdong Key Laboratory of Agricultural Products Processing, Guangzhou 510610, China; 19174160836@163.com (L.Y.); 17336427607@163.com (P.L.); 19878713326@163.com (T.Z.); zhangyhgx@163.com (Y.Z.)

**Keywords:** cyclophosphamide, premature ovarian failure, urolithin A, oxidative stress, hypothalamic-pituitary-ovarian axis, functional food strategy

## Abstract

**Highlights:**

- CTX induced a POF-like phenotype with follicular atresia and endocrine disruption.
- CoQ10 and the designed formulations (Base and Base + U) improved cyclicity, ovarian histology, and hormone profiles.
- The treatments alleviated ovarian oxidative-inflammatory stress (GSH-Px↑, MDA↓, cytokines↓).
- Ovarian functional transcripts were restored (*Fshr*, *Esr1*, *Hsd17b2*) by the treatments.
- Urolithin A addition (Base + U) showed an incremental benefit on lipid peroxidation/inflammation.

**Abstract:**

Cyclophosphamide (CTX)-induced premature ovarian failure (POF) is characterized by disruption of the follicular microenvironment, granulosa-cell loss, endocrine imbalance, and oxidative-inflammatory injury. Here, we evaluated two multi-bioactive formulations developed to enhance ovarian stress resilience: a base formulation containing coenzyme Q10, calcium L-5-methyltetrahydrofolate, and *Vitex agnus-castus* extract (Base), and a urolithin A-enriched formulation (Base + U). Using a CTX-induced female C57BL/6 mouse model, we integrated phenotypic, histological, endocrine, oxidative-inflammatory, and transcriptional readouts to assess efficacy and mechanistic consistency. CTX markedly reduced ovarian index, disrupted estrous cyclicity, shifted follicle development toward atresia, increased granulosa-cell apoptosis, and caused endocrine dysregulation, including decreased anti-Müllerian hormone and estradiol and increased GnRH, FSH, and LH. CoQ10, Base, and Base + U each partially alleviated these abnormalities, improving ovarian index and coat condition, showing a trend toward improved follicular distribution, and normalizing hormone profiles. CTX also induced an ovarian oxidative-inflammatory shift, as reflected by decreased GSH-Px, increased MDA, and elevated IL-1β, IL-6, and TNF-α, all of which were attenuated by the interventions. Notably, Base + U more effectively reduced lipid peroxidation and TNF-α than Base alone. Consistently, ovarian transcripts related to follicle responsiveness and steroid regulation, including *Fshr*, *Esr1*, and *Hsd17b2*, were restored, whereas hypothalamic qRT-PCR analysis did not reveal robust transcriptional alterations within the intervention window. These findings suggest that the urolithin A-enhanced formulation partially alleviates CTX-induced ovarian dysfunction by suppressing oxidative-inflammatory stress and preserving granulosa-cell and follicle fate.

## 1. Introduction

Premature ovarian failure (POF) is characterized by diminished ovarian reserve, disrupted cyclicity, and endocrine imbalance driven by impaired hypothalamic-pituitary-ovarian (HPO) axis feedback [1]. Beyond fertility loss, ovarian endocrine decline affects systemic physiology and health trajectories [2]. Given the ovary’s high sensitivity to cellular stressors, clarifying stress-linked mechanisms of ovarian dysfunction and identifying safe, multi-target interventions remain important both biologically and translationally [3].

Cyclophosphamide (CTX) is widely used in chemotherapy, yet its gonadotoxicity represents a major concern [4]. In experimental models, CTX exposure recapitulates key features of POF, including ovarian atrophy, follicular loss, increased follicular atresia, and broad hormone dysregulation [5]. Mechanistically, CTX-associated ovarian injury is increasingly understood as a microenvironmental failure state in which oxidative damage, inflammatory activation, and stress-responsive cell death programs converge to compromise granulosa-cell (GC) viability and steroidogenic competence [6,7]. Because GC survival and function are essential for follicle development and ovarian steroid output, stress-driven GC dysfunction provides a plausible mechanistic bridge linking local ovarian injury to systemic endocrine collapse and compensatory hypergonadotropism [7].

Nutritional interventions with antioxidant and anti-inflammatory properties hold promise for mitigating such gonadotoxic effects [8]. From a functional food and nutraceutical perspective, these observations motivate a strategy that targets stress containment as an upstream lever for preserving ovarian competence. However, many nutritional interventions rely on a narrow set of endpoints, which hinders a unified, axis-resolved interpretation spanning ovarian microenvironmental stress, downstream GC/follicle fate decisions, steroidogenic competence, and HPO-axis feedback control. A more integrated framework is needed to provide mechanistic credibility and guide translation toward multi-bioactive formulations designed to modulate redox-inflammatory networks and cellular resilience.

Here, we developed and evaluated two proprietary formulations with a clear mechanistic design rationale. The first formulation (Base) combines coenzyme Q10 (CoQ10) [9], calcium L-5-methyltetrahydrofolate [10], and *Vitex agnus-castus* extract [11], aiming to support mitochondrial/redox homeostasis and endocrine-related ovarian responsiveness through complementary nutritional nodes. Among them, CoQ10 is an endogenous lipid-soluble quinone involved in mitochondrial electron transport and redox homeostasis. Calcium L-5-methyltetrahydrofolate is the biologically active form of folate and participates in one-carbon metabolism and methylation-related physiological processes. *Vitex agnus-castus* extract, commonly known as chasteberry extract, is a botanical ingredient that has been clinically investigated in women’s health contexts, particularly premenstrual syndrome, cyclic mastalgia, and luteal-phase defects associated with latent hyperprolactinemia [12,13]. In the present study, *Vitex agnus-castus* extract was used as a proprietary raw material within the Base formulation rather than as a standalone commercial product. Thus, the Base formulation was designed to integrate mitochondrial/redox support, methylation-related nutritional support, and endocrine-related botanical modulation. The second formulation (Base + U) builds upon Base by incorporating urolithin A (UA), a diet-derived gut microbial metabolite associated with enhanced cellular stress tolerance and mitochondrial quality control [14,15]. We hypothesized that adding UA could provide an incremental protective advantage under CTX challenge by strengthening ovarian stress containment and reducing pro-apoptotic bias [16], thereby improving follicle fate and endocrine feedback restoration.

To test these formulations and resolve mechanism-to-function alignment, we employed a CTX-induced POF mouse model and implemented a multi-level evaluation spanning: (i) general phenotypes (body weight, food intake, ovarian index, coat condition), (ii) estrous-cycle regularity, (iii) ovarian histoarchitecture and GC apoptosis, (iv) comprehensive serum hormone profiles reflecting ovarian output and HPO-axis feedback, (v) ovarian oxidative stress and inflammatory cytokines, and (vi) gene-expression signatures in ovary and hypothalamus. This design enables us to determine not only whether Base and Base + U improve CTX-induced ovarian dysfunction, but also whether their efficacy aligns with an ovary-centered sequence in which stress mitigation is followed by stabilization of GC/follicle fate, restoration of steroidogenic competence, and normalization of endocrine feedback, thereby providing a stronger theoretical basis for functional food translation.

## 2. Materials and Methods

### 2.1. Materials

CoQ10, UA, *Vitex agnus-castus* extract and calcium L-5-Methyltetrahydrofolate were provided by Shenzhen Porshealth Bioengineering Co., Ltd. (Shenzhen, China). CTX (product no. C0768-5G) was purchased from Merck Sigma Aldrich (Shanghai) Trading Co., Ltd. (Shanghai, China). The following commercial ELISA kits were used: testosterone (T) from Elabscience Biotechnology Co., Ltd. (Wuhan, China); estradiol (E2), luteinizing hormone (LH), cyclic adenosine monophosphate (cAMP), gonadotropin-releasing hormone (GnRH), interleukin-1β (IL-1β), and tumor necrosis factor-α (TNF-α) from Coibo Biotechnology Co., Ltd. (Shanghai, China); follicle-stimulating hormone (FSH) from Jianglai Biotechnology Co., Ltd. (Shanghai, China); and anti-Müllerian hormone (AMH) from Wuhan Huamei Biotech Co., Ltd. (Wuhan, China). Assay kits for malondialdehyde (MDA), superoxide dismutase (SOD), glutathione peroxidase (GSH-Px), and interleukin-6 (IL-6) were supplied by Nanjing Jiancheng Bioengineering Institute Co., Ltd. (Nanjing, China). All other reagents used in the experiments were of analytical grade.

### 2.2. Study Formulations

For this study, two proprietary formulations were utilized and are referred to by their research codes throughout the manuscript. CoQ10 and UA were provided as individual active ingredients. The Base and Base + U samples were provided as defined proprietary formulation mixtures. Base contained CoQ10, calcium L-5-methyltetrahydrofolate, and *Vitex agnus-castus* extract, whereas Base + U was based on the Base formulation with the addition of UA. In the animal experiment, the CoQ10 group received CoQ10 at 16.67 mg/kg, while the Base and Base + U groups received the corresponding total formulation at 41.67 mg/kg. The exact quantitative composition, component ratios, and preparation method of the proprietary formulations are not disclosed due to commercial confidentiality.

### 2.3. Animals and Treatment

Female C57BL/6 mice (8–10 weeks old) were housed under specific pathogen-free (SPF) conditions with a 12 h light/dark cycle, controlled temperature (22 ± 2 °C) and humidity (50 ± 10%), and were provided with food and water ad libitum. Female C57BL/6 mice were selected because this strain is widely used in reproductive toxicology and CTX-induced ovarian dysfunction models, allowing comparison with previous studies. Mice were maintained on a standard laboratory chow diet throughout the experiment. All animal procedures were approved by the Laboratory Animal Ethics Committee of Shenzhen Rongwan Biomedical Laboratory Animal Center (Approval No. RW-IACUC-24-0082). After one week of acclimatization, the mice were randomly divided into five groups (*n* = 9 per group; see Figure S1): the Normal Control group received daily intraperitoneal (i.p.) injection of saline along with daily oral saline gavage for 6 weeks; the Model group was injected i.p. with CTX (100 mg/kg) daily for 2 weeks to establish the POF model, followed by daily oral saline gavage for a total of 6 weeks; the Positive Control (CoQ10) group received the same CTX regimen and then daily oral gavage of CoQ10 (16.67 mg/kg) for 6 weeks; the Base treatment group was given the same CTX injections followed by daily oral gavage of the Base formulation (41.67 mg/kg) for 6 weeks; and the Base + U treatment group underwent the same CTX regimen followed by daily oral gavage of the Base + U formulation (41.67 mg/kg) for 6 weeks. Throughout the experiment, body weight and food intake were measured and recorded every 5 days. At the end of the experimental period, mice were anesthetized via intraperitoneal injection of 4% chloral hydrate at a dose of 0.2 mL per 20 g body weight. Blood samples were subsequently collected from the orbital sinus. Following blood collection, mice were immediately euthanized by cervical dislocation, and ovarian and hypothalamic tissues were rapidly dissected and collected for further analysis. Hypothalamic tissues were dissected from the ventral diencephalic region using anatomical landmarks based on the mouse brain atlas. Briefly, after brain removal, the hypothalamic block was isolated from the ventral surface. The rostral boundary was defined by the optic chiasm, the caudal boundary by the mammillary bodies, and the lateral boundaries by the optic tracts/hypothalamic sulci. Tissue collection was performed under a stereomicroscope by trained personnel under consistent experimental conditions. No independent histological or molecular validation of dissection purity was performed.

### 2.4. Evaluation of Ovarian Index

Following dissection, both ovaries were carefully excised and weighed. The ovarian index, expressed as a percentage, was determined using the following formula:

$$Ovarian\;Index=\frac{Weight\;of\;both\;ovaries\;(mg)}{Body\;Weight\;(g)}\times 100\%$$

### 2.5. Vaginal Cytology and Estrous Cycle Staging

Vaginal smears were collected daily at 10:00 a.m. for 10 consecutive days beginning from the start of the modeling procedure. Briefly, 10 μL of normal saline was flushed and aspirated 2–3 times from the vaginal orifice (insertion depth ~0.5 cm) using a 20 μL micropipette. The aspirate was smeared onto a glass slide, air-dried, stained with hematoxylin and eosin (H&E), and examined microscopically for estrous cycle staging. Estrous cycle stages were identified according to vaginal cytology. Proestrus was characterized predominantly by nucleated epithelial cells. Estrus was characterized mainly by cornified squamous epithelial cells. Metestrus was identified by the presence of mixed leukocytes, cornified epithelial cells, and nucleated epithelial cells, whereas diestrus was characterized by a predominance of leukocytes. Follicle classification criteria were according to Hirshfield and Midgley [17].

### 2.6. Serum Hormone Measurement

Blood was collected from the orbital sinus post-intervention. After clotting (2 h, RT), samples were centrifuged (4000× *g*, 15 min, 4 °C). Serum was aliquoted and stored at −80 °C. Levels of T, E2, LH, FSH, AMH, cAMP, and GnRH were measured using commercial ELISA kits (see Section 2.1) according to the manufacturer’s protocols.

### 2.7. Histological Analysis

Left ovaries were collected and fixed in 4% paraformaldehyde at the experimental endpoint. Tissues were processed through dehydration, embedded in paraffin, and sectioned. Sections were deparaffinized, stained with Mayer’s hematoxylin (5–7 min), rinsed in water for bluing, air-dried, and mounted with neutral balsam. Ovarian morphology was examined under a light microscope. Atretic follicles were identified based on typical morphological features, including disorganized granulosa-cell layers, pyknotic nuclei, granulosa-cell detachment, irregular follicular structure, and oocyte degeneration. Healthy follicles were characterized by an intact oocyte, organized granulosa-cell layers, and preserved follicular architecture.

### 2.8. Terminal Deoxynucleotidyl Transferase dUTP Nick-End Labeling (TUNEL) Assay

Ovarian cell apoptosis was assessed by TUNEL. Sections were fixed in 4% paraformaldehyde (1 h), incubated with 3% H_2_O_2_ (10 min), and permeabilized with 0.1–0.5% Triton X-100 (5 min). Apoptosis was detected using an cell death detection kit, and nuclei were counterstained with DAPI (8 min). TUNEL-positive cells were visualized under an Olympus BX61 fluorescence microscope (Olympus Corporation, Tokyo, Japan), and the apoptosis rate was quantified with ImageJ software (version 1.54f, National Institutes of Health, Bethesda, MD, USA).

### 2.9. Quantitative Real-Time PCR (qRT-PCR)

For qRT-PCR analysis, one-half of the left ovary and the hypothalamus were collected separately. Tissues from every three mice within the same group were pooled to generate one biological replicate. Thus, three pooled biological replicates were obtained for each group and each tissue type. Total RNA was extracted from pooled whole ovarian tissues and pooled hypothalamic tissues using TRIzol reagent (Takara Bio Inc., Shiga, Japan). After assessing concentration and purity using a spectrophotometer (NanoDrop 2000c, Thermo Fisher Scientific, Waltham, MA, USA), cDNA was synthesized using a reverse transcription kit (Takara Bio Inc., Shiga, Japan). qRT-PCR was performed on a StepOne system (Applied Biosystems, Foster City, CA, USA) with SYBR Green (Takara Bio Inc., Shiga, Japan). Primer sequences are listed in Supplementary Table S1. Gene expression was analyzed using the 2^−ΔΔCT^ method.

### 2.10. Oxidative Stress and Inflammatory Marker Assays

Frozen right ovarian tissue (0.15–0.2 g) was homogenized in 0.9% saline (1:9, *w*/*v*) to yield a 10% homogenate. After double centrifugation (13,000× *g*, 10 min each), the supernatant was collected for analysis. Following the manufacturers’ instructions, MDA content was measured at 532 nm, while SOD and GSH-Px activities, as well as IL-6, IL-1β, and TNF-α levels, were determined at 560 nm using commercial assay kits (see Section 2.1).

### 2.11. Statistical Analysis

Data are presented as mean ± SEM unless otherwise stated. Differences among groups were analyzed by one-way ANOVA followed by Duncan’s test. *p* < 0.05 was considered statistically significant.

## 3. Results

### 3.1. Effects of Treatments on the General Health of Mice with CTX-Induced POF

**Figure 1:**
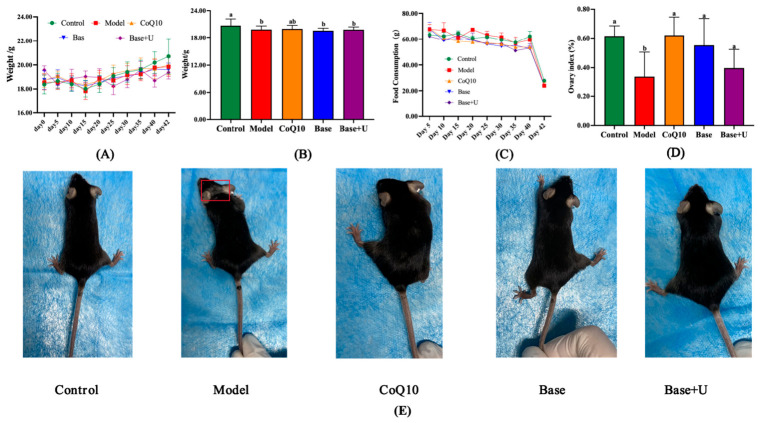
Effects of treatments on the general health and ovarian index of mice with CTX-induced POF. (**A**) Body weight changes throughout the experimental period. (**B**) Terminal body weight before sacrifice. (**C**) Average food intake per measurement interval. (**D**) Ovarian index, calculated as the ovary weight/body weight ratio. (**E**) Representative images depicting the coat condition of mice. Red boxes indicate representative regions of visible hair loss or coat deterioration in the CTX-treated model group. Data are presented as mean ± SEM. Different lowercase letters above bars or data points indicate significant differences among groups; groups sharing the same letter are not significantly different, whereas groups with different letters differ significantly at *p* < 0.05. Note: The apparent decrease in food intake from Day 40 to Day 42 is due to a shortened recording interval of 2 days compared with the standard 5-day interval.

CTX, a widely employed chemotherapeutic agent, is extensively used in the treatment of various cancers [18]. However, its detrimental effects on reproductive health, particularly ovarian function, have become a significant concern. Research has demonstrated that CTX administration can successfully establish a mouse model of POF, characterized by alterations in ovarian index and hair growth [19]. As illustrated in Figure 1A, mice in the CTX-treated model group exhibited a reduction in body weight compared with the control group, despite no significant change in food intake. At the end of the experiment, the average body weight of CTX-treated mice was 0.86 g lower than that of the control mice. This weight loss was not significantly reversed by CoQ10, Base, or Base + U treatment (Figure 1A–C). Crucially, the ovarian index, a key parameter, was markedly lower in the CTX group than in controls, confirming the successful induction of the POF model. Notably, co-treatment with CoQ10, Base, and Base + U significantly attenuated this decrease in ovarian index (Figure 1D, *p* < 0.05). Furthermore, visible coat deterioration and hair loss, particularly in the head and shoulder regions, were observed in the CTX-treated model group, as indicated by the representative images and red boxes in Figure 1E. Compared with the model group, the treatment groups showed a partial improvement in coat condition; however, this observation was used only as representative visual support and was not considered quantitative evidence.

### 3.2. Effects of Treatments on Restoration of Estrous Cycle Regularity in CTX-Induced POF Model Mice

**Figure 2:**
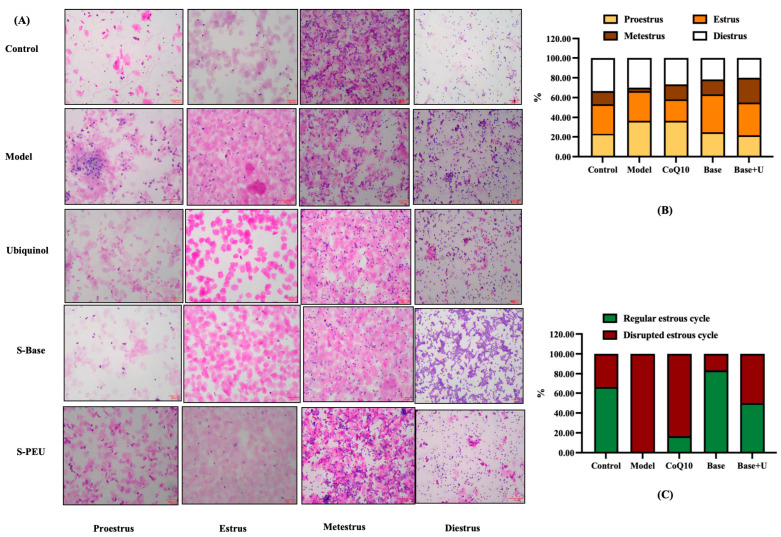
Restoration of estrous cycle regularity by treatments in POF model mice. (**A**) Representative micrographs of vaginal cytology across different estrous stages. Scale bar: 100 μm. (**B**) Proportional distribution of each estrous cycle stage among groups. (**C**) Percentage of mice exhibiting regular versus disrupted estrous cycles. Proestrus, estrus, metestrus, and diestrus were identified based on the cytological features described in Section 2.5.

A key pathological feature of the CTX-induced POF model is the disruption of the murine estrous cycle [20]. As shown in Figure 2A,B, compared with the control group, the model group exhibited a prolonged duration of proestrus, alongside extended phases of estrus, metestrus, and diestrus. These collective alterations confirm a significant disturbance in cyclicity (Figure 2C).

### 3.3. Effects of Treatments on Ovary Dysfunction in POF Mice

**Figure 3:**
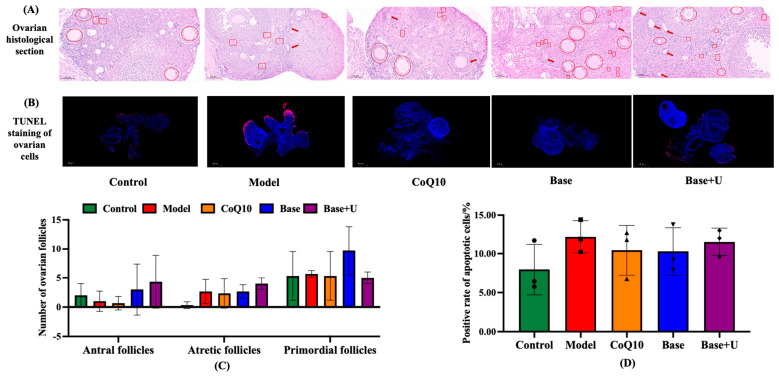
Treatment effects on ovarian histoarchitecture and granulosa cell apoptosis in POF mice. (**A**) Representative hematoxylin and eosin (H&E)-stained sections of ovarian tissues. (**B**) Representative images of TUNEL staining of ovarian sections. Red fluorescence indicates TUNEL-positive (apoptotic) cells; blue fluorescence (DAPI) labels cell nuclei. (**C**) Quantitative analysis of follicle counts at different developmental stages. (**D**) Quantitative analysis of ovarian cell apoptosis rates. No statistically significant differences were observed among groups for antral, atretic, or primordial follicle counts in panel C or for apoptosis rates in panel D. In panel A, boxes highlight primordial follicles; arrows indicate atretic follicles; circles denote mature follicles. Scale bar: 100 μm. TUNEL-positive cells were quantified from multiple microscopic fields, and representative images are shown.

Ovarian reserve, indicative of the ovary’s endocrine and reproductive capacity, is determined by the size and quality of the primordial follicle pool and its developmental potential into mature follicles [19]. Representative hematoxylin and eosin (HE)-stained ovarian sections are shown in Figure 3A. Ovaries in the CTX model group predominantly contained atretic follicles, whereas other groups exhibited relatively greater numbers of primordial and mature follicles. Quantitative follicle analysis (Figure 3C) revealed no statistically significant differences in total follicle counts across groups. Nevertheless, the model group showed a tendency toward reduced mature/antral follicles and increased atretic follicles, whereas CoQ10, Base, and Base + U treatment showed a trend toward improved follicular distribution. Base treatment also showed a tendency to preserve primordial follicles. The relatively moderate improvement in follicular parameters may be attributed to two factors: the intervention duration may have been insufficient to fully manifest follicular restoration, and the primary therapeutic effects may currently be focused on hormonal modulation rather than direct follicular development.

### 3.4. Effects of Treatments on Granulosa-Cell Apoptosis in POF Mice

Granulosa cells (GCs) are essential for follicular development, performing key functions including the aromatization of androgens to estrogens, progesterone synthesis, and secretion of factors such as insulin-like growth factor (IGF) [21]. Pathological insults can trigger GC apoptosis and senescence, leading to follicular atresia, accelerated oocyte depletion, and ultimately ovarian dysfunction [22]. As shown in Figure 3B, TUNEL staining showed increased ovarian apoptotic signals in the CTX model group compared with the control group. TUNEL-positive cells were also detectable in the CoQ10, Base, and Base + U treatment groups, but their overall abundance appeared lower than that in the CTX model group. Consistent with the representative images, the quantitative analysis in Figure 3D showed a downward trend in apoptosis rate after treatment, although the intergroup differences did not reach statistical significance.

### 3.5. Effects of Treatments on Serum Hormone Levels in POF Mice

**Figure 4:**
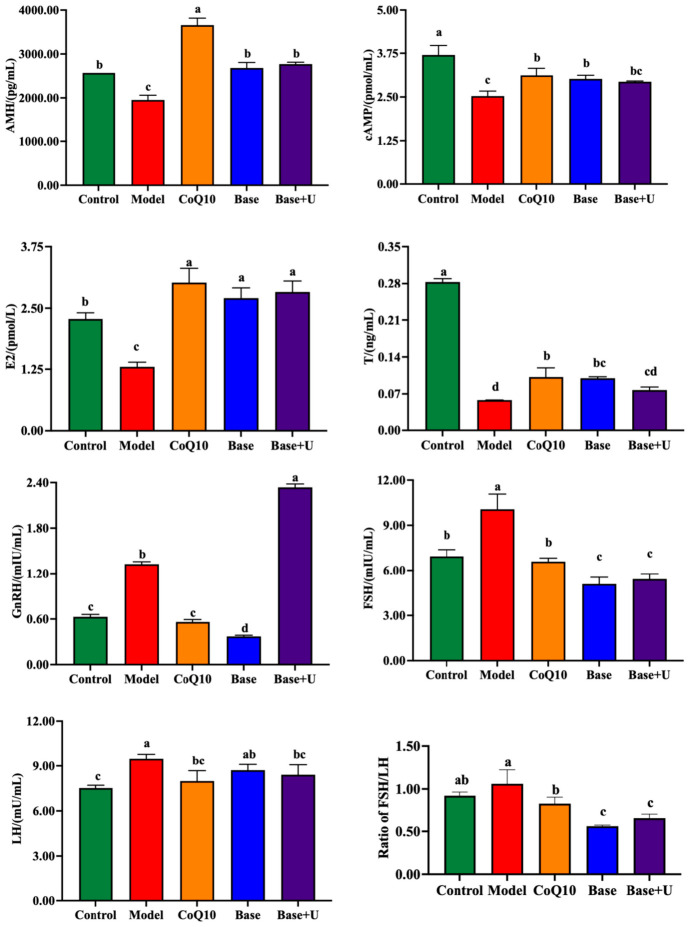
Effects of treatments on serum hormone levels in POF mice (*n* = 4). Data are presented as mean ± SEM. Statistical differences among groups were analyzed by one-way ANOVA followed by Duncan’s test. Different lowercase letters (a, b, c, d) above bars denote statistically significant differences among groups (*p* < 0.05).

Serum hormone levels are key indicators of ovarian function [23]. In particular, AMH serves as a reliable marker of ovarian reserve [24]. As shown in Figure 4, AMH levels were significantly lower in the CTX group compared to controls (*p* < 0.05). CoQ10 treatment markedly increased AMH levels, with Base and Base + U also showing significant upregulation (*p* < 0.05). Similar remarkable restorative trends were observed for E2, cAMP, and T (all *p* < 0.05). Moreover, CTX-induced elevations in GnRH, FSH, LH, and the FSH/LH ratio were significantly reduced by CoQ10, Base, and Base + U treatments (*p* < 0.05). Together, these findings indicate that all three treatments positively modulate hormone dysregulation in POF mice, thereby ameliorating ovarian dysfunction (Figure 3).

### 3.6. Effects of the Treatments on Ovarian Oxidative Stress and Inflammatory Markers in POF Mice

**Figure 5:**
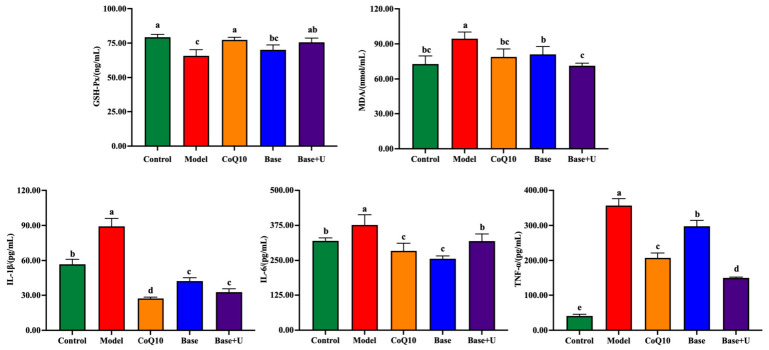
Effects of the treatments on ovarian oxidative stress and inflammatory markers in POF mice (*n* = 4). Data are presented as mean ± SEM. Different lowercase letters (a, b, c, d, e) above bars denote statistically significant differences among groups (*p* < 0.05).

The dysregulation of serum sex hormones, indicative of ovarian dysfunction, is likely mediated by oxidative and inflammatory injury [3,25]. As shown in Figure 5, CTX treatment significantly decreased the GSH-Px level from 79.27 ± 2.07 ng/mL (control) to 65.78 ± 4.43 ng/mL (*p* < 0.05). This reduction was effectively reversed by CoQ10 (77.30 ± 1.91 ng/mL, *p* < 0.05), Base (70.10 ± 3.57 ng/mL, *p* < 0.05), and Base + U (75.59 ± 3.04 ng/mL, *p* < 0.05). Concurrently, the elevated MDA content in the model group (94.43 ± 5.74 nmol/mL) was significantly inhibited by all treatments, with Base + U showing the most potent effect (71.28 ± 2.25 nmol/mL, *p* < 0.05). Furthermore, CTX administration significantly upregulated pro-inflammatory cytokines, including IL-1β, IL-6, and TNF-α (*p* < 0.05), indicating a state of inflammatory stress in the ovary [26]. All treatments attenuated this inflammatory response to varying degrees. Notably, CoQ10 was most effective in reducing IL-1β (*p* < 0.05), Base showed the greatest suppression of IL-6 (*p* < 0.05), and Base + U exhibited the strongest downregulation of TNF-α, lowering its level from 356.78 pg/mL to 149 pg/mL (*p* < 0.05). These results demonstrate that each treatment can ameliorate the impaired antioxidant and inflammatory systems in CTX-induced POF mice.

### 3.7. Effects of Treatments on the Expression of Ovarian Function-Related Genes in POF Mice

**Figure 6:**
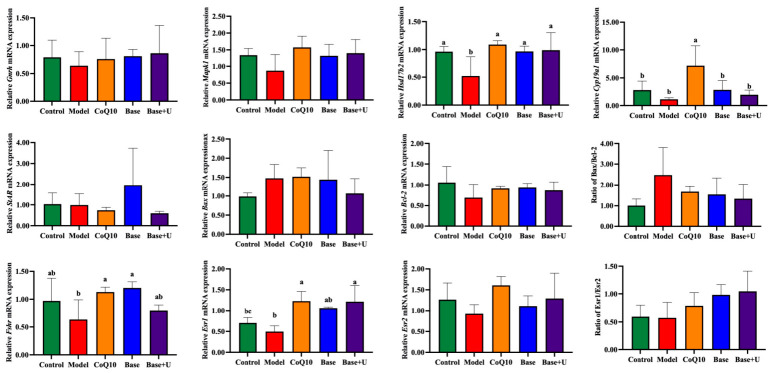
Effects of treatments on the expression of ovarian function-related genes in POF mice. Data were obtained from pooled whole ovarian tissue samples, with three pooled biological replicates per group (*n* = 3). Statistical differences among groups were analyzed by one-way ANOVA followed by Duncan’s test. Statistical letters are shown only for panels with significant intergroup differences. No statistical letters are shown for panels without significant differences. Groups sharing the same letter are not significantly different, whereas groups with different letters differ significantly at *p* < 0.05.

Gene expression profiling offers critical insights into the molecular underpinnings of ovarian function [27]. To elucidate the underlying mechanisms of ovarian dysfunction, we analyzed the expression of key ovarian function-related genes in POF mice (Figure 6). CTX treatment significantly downregulated the expression of *Hsd17b2*, *Fshr*, and *Esr1*, all of which were restored by CoQ10, Base, and Base + U treatments (*p* < 0.05). For the remaining ovarian function- and apoptosis-related genes, including *Cyp19a1*, *Esr2*, *StAR*, *GnRH*, *Bax*, *Bcl-2*, and the Bax/Bcl-2 ratio, no consistent statistically significant intergroup differences were detected. Therefore, these nonsignificant gene-expression data are presented to provide a complete overview of the ovarian transcriptional profile but are not interpreted as treatment-induced regulatory effects. It should be noted that these qRT-PCR data were obtained from pooled whole ovarian tissue and therefore reflect overall ovarian transcript changes rather than granulosa-cell-specific expression.

### 3.8. Effects of Treatments on the Expression of Hypothalamic Reproductive Regulatory Genes in POF Mice

**Figure 7:**
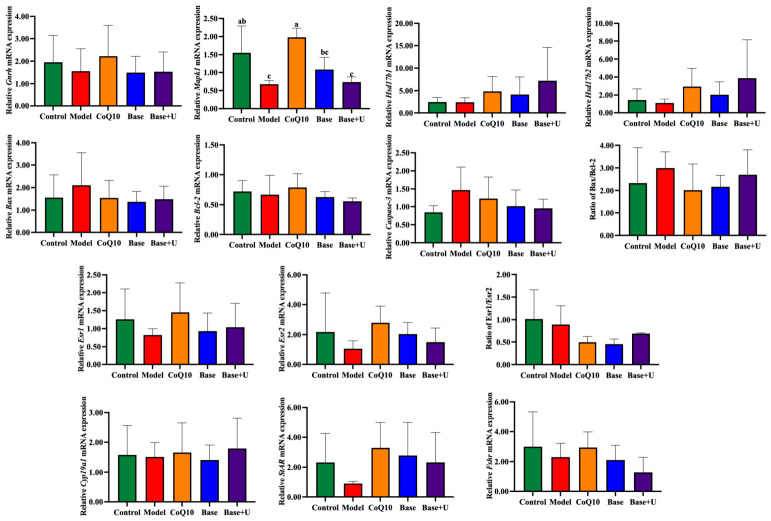
Effects of treatments on the expression of hypothalamic reproductive regulatory genes in POF mice. Data were obtained from pooled hypothalamic tissue samples, with three pooled biological replicates per group (*n* = 3). Statistical letters are shown only for panels with significant intergroup differences. No statistical letters are shown for panels without significant differences. Groups sharing the same letter are not significantly different, whereas groups with different letters differ significantly at *p* < 0.05.

Recent studies have highlighted the modulation of the HPO axis as a key mechanism for improving ovarian function [28]. To investigate whether this pathway is involved in our model, we analyzed the expression of HPO axis-related genes in CTX-induced POF mice. As shown in Figure 7, among the genes examined, only *Mapk1* mRNA expression was significantly decreased in the CTX-treated model group and was significantly restored by CoQ10 treatment (*p* < 0.05). For the other hypothalamic reproductive regulatory and apoptosis-related genes, including *Bax*, *Caspase-3*, *Bcl-2*, *Esr1*, *Esr2*, *StAR*, *Fshr*, and the Bax/Bcl-2 ratio, no robust statistically significant intergroup differences were detected. These nonsignificant panels were retained to show the complete hypothalamic transcriptional profile but were not interpreted as evidence of treatment-induced central transcriptional regulation.

## 4. Discussion

**Figure 8:**
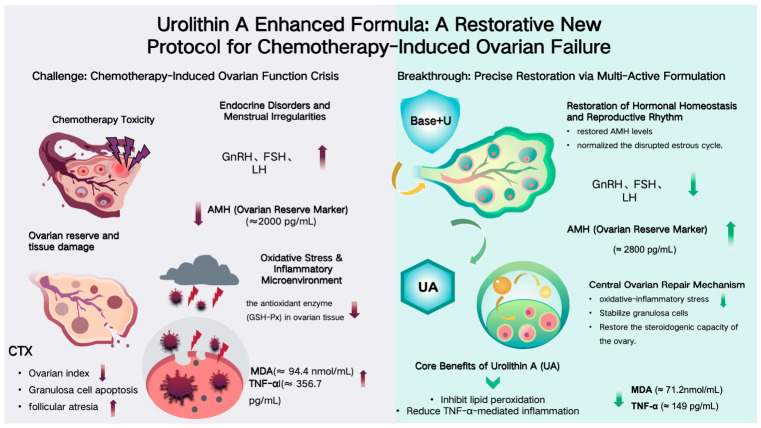
Schematic summary of the underlying mechanisms by which the sample formulation ameliorates CTX-induced POF. Purple arrows indicate CTX-induced pathological changes relative to the control condition, whereas green arrows indicate Base + U/UA-mediated restorative effects or normalization relative to the CTX model. Upward and downward arrows denote increases and decreases, respectively. The gray/pink left panel represents chemotherapy-induced ovarian dysfunction, while the light green right panel represents the restorative intervention state.

In this study, we established a robust CTX-induced POF model featuring ovarian atrophy, estrous-cycle disruption, follicular fate imbalance, granulosa-cell (GC) apoptosis, broad endocrine dysregulation, and an ovarian oxidative-inflammatory stress state [29]. Across multiple phenotypic layers, CoQ10, Base, and Base + U each partially counteracted this CTX-driven failure cascade, improving ovarian index and coat condition (Figure 1C,D), restoring estrous cyclicity (Figure 2), preserving ovarian histoarchitecture with a shift toward more mature follicles and fewer atretic follicles (Figure 3A,C), normalizing multi-hormone profiles (Figure 4), and alleviating ovarian oxidative stress and inflammatory cytokine burden (Figure 5). Consistent changes in ovarian functional transcripts further supported improvement in receptor/steroidogenic competence (Figure 6), whereas hypothalamic qRT-PCR analysis did not reveal broad transcriptional alterations within the current intervention window (Figure 7). Collectively, these findings are consistent with an ovarian-centered mechanism whereby reducing ovarian oxidative-inflammatory stress helps stabilize GC/follicle fate and restore endocrine feedback, as summarized schematically in Figure 8.

As an alkylating chemotherapeutic agent, CTX is closely associated with cancer treatment-related gonadal toxicity. Cancer treatment-related ovarian dysfunction has important implications for women of childbearing potential, including menstrual abnormalities, impaired fertility, and long-term endocrine and systemic health consequences [30]. In experimental and preclinical contexts, CTX-induced ovarian toxicity has been linked to oxidative stress, inflammatory activation, and apoptosis, and antioxidant-based interventions have been explored as potential strategies to mitigate ovarian injury [31]. Although the present study focuses on ovarian dysfunction, CTX-related gonadotoxicity is also relevant to male reproductive health, as CTX has been reported to impair male gonadal function by damaging germ cells, Leydig cells, and Sertoli cells and disrupting spermatogenesis and androgen-related regulation [32]. Compared with previous studies focusing on single antioxidants or isolated ovarian endpoints, the present study integrates ovarian histology, endocrine profiles, oxidative-inflammatory markers, and ovarian/hypothalamic transcriptional readouts to evaluate a UA-enhanced multi-bioactive formulation strategy.

A central question is whether ovarian oxidative stress and inflammation are merely accompanying phenomena or represent an upstream driver of functional collapse [20,33]. CTX induced a pronounced oxidative-inflammatory shift in ovarian tissue, reflected by reduced antioxidant capacity (GSH-Px) and increased lipid peroxidation (MDA) [3], together with marked elevations of pro-inflammatory cytokines (IL-1β, IL-6, TNF-α) (Figure 5). This ovarian microenvironment is biologically positioned to compromise GC survival and steroidogenic competence, thereby biasing follicle fate toward atresia and reducing ovarian endocrine output [34,35,36]. In line with this framework, all three interventions attenuated oxidative-inflammatory injury (Figure 5), and this upstream improvement coherently aligned with downstream rescue signals at the histological, endocrine, and transcriptional levels (Figure 3, Figure 4, Figure 5 and Figure 6). These findings are consistent with, but do not prove, a model in which ovarian oxidative-inflammatory stress contributes to GC dysfunction, follicular fate imbalance, and endocrine disruption under CTX challenge.

Although CTX reduced body weight without significantly altering food intake (Figure 1A,B), none of the interventions restored body weight, indicating limited impact on global CTX-associated wasting under these conditions. Importantly, ovarian-specific outcomes improved despite this, most notably the ovarian index (Figure 1C) and coat condition (Figure 1D), suggesting that ovarian protection can be achieved without broad systemic normalization. This decoupling strengthens the inference that the treatments act through local ovarian stress and functional pathways, rather than reflecting generalized health recovery.

Ovarian histology indicated that CTX exposure perturbed follicular fate, with ovaries dominated by atretic follicles and reduced representation of healthy developing structures (Figure 3A). Quantitative follicle staging showed no statistically significant differences among groups for antral, atretic, or primordial follicle counts. Nevertheless, CTX treatment tended to shift the follicular distribution toward fewer mature/antral follicles and more atretic follicles, whereas Base and Base + U showed a trend toward increased mature/antral follicle numbers. Base treatment also showed a tendency to preserve primordial follicles (Figure 3C). These changes are biologically meaningful even when total follicle numbers do not differ dramatically, because folliculogenesis is time-structured and early rescue can manifest as reduced ongoing atresia and improved maturation competence rather than immediate expansion of the entire follicle pool [37].

GC apoptosis represents a key intermediate link between microenvironmental stress and follicular atresia [27]. CTX markedly increased TUNEL-positive cells in ovarian tissue (Figure 3B), and apoptosis quantification showed a downward trend under CoQ10, Base, and Base + U (Figure 3D). While the inter-treatment differences in apoptosis did not uniformly reach statistical significance, the directionality is consistent with the observed improvement in follicular fate (Figure 3C) and with the strong stress-mitigation phenotype (Figure 5), together supporting a model in which dampening ovarian stress reduces GC attrition and limits atresia.

The multi-hormone panel provides the most internally coherent evidence for system-level improvement. CTX induced a typical endocrine failure signature: reduced AMH and steroid-related readouts alongside elevations of GnRH, FSH, LH and an increased FSH/LH ratio (Figure 4), consistent with impaired ovarian output and compensatory upstream drive. CoQ10, Base, and Base + U each partially normalized this signature in a coordinated manner—raising AMH and restoring steroidogenic-associated parameters while suppressing hypergonadotropism (Figure 4). Importantly, this pattern is best interpreted as recovery of ovarian endocrine competence and reconstruction of negative feedback, rather than isolated modulation of a single hormone [38,39]. In this context, the improved estrous cyclicity under Base and Base + U (Figure 2B,C) provides a functional readout of recovered reproductive rhythmicity that aligns with endocrine normalization.

To bridge phenotype to mechanism, we assessed ovarian function-related transcripts. CTX downregulated key genes including *Fshr* and *Esr1* (Figure 6), both critical for GC responsiveness and estrogen signaling integration within the follicle [40,41]. CoQ10, Base, and Base + U restored these significantly altered transcripts (Figure 6), providing molecular support for improved ovarian tissue-level responsiveness. In contrast, several other apoptosis- and receptor-balance-related indices did not show consistent statistically significant intergroup differences and therefore were not interpreted as treatment-specific regulatory effects [42,43,44]. These observations provide a broad ovarian transcriptional context but do not, by themselves, establish a distinct cell-type-specific pathway.

Because pooled whole ovarian tissue was used for qRT-PCR, the present data cannot distinguish granulosa-cell-, theca-cell-, stromal-, or oocyte-specific transcriptional responses. Therefore, the changes in ovarian function-related genes should be interpreted as overall ovarian tissue-level alterations rather than granulosa-cell-specific regulation. In addition to granulosa cells, the theca compartment may also be affected by CTX treatment, potentially through altered androgen production, stromal remodeling, or disrupted follicular paracrine communication. Since theca cells provide androgen substrates for granulosa-cell estrogen synthesis and contribute to follicular microenvironment regulation, changes in the theca compartment may also participate in CTX-induced ovarian dysfunction. Future studies using isolated granulosa and theca cells, laser-capture microdissection, or single-cell transcriptomics will be required to define cell-type-specific mechanisms.

In the hypothalamus, the present qRT-PCR analysis did not reveal broad or robust transcriptional alterations within the current intervention window. Among the genes examined, only Mapk1 showed a significant decrease in the CTX-treated model group and restoration by CoQ10 treatment (Figure 7), whereas the remaining genes did not show robust statistically significant intergroup differences. Therefore, the current data should not be interpreted as evidence of broad central transcriptional remodeling. Given the small size and anatomical complexity of the mouse hypothalamus, the hypothalamic qRT-PCR results should be interpreted with caution. Although tissues were collected by trained personnel according to anatomical landmarks under consistent conditions, future studies using more spatially resolved and cell-type-resolved approaches, such as validated microdissection, immunofluorescent detection of hypothalamic releasing factors, bulk RNA-seq, or single-cell RNA-seq, would provide a more robust assessment of central HPO-axis regulation [45,46].

While CoQ10 served as a reference intervention, both proprietary formulations produced convergent rescue across multiple outcome layers (Figure 1, Figure 2, Figure 3, Figure 4, Figure 5 and Figure 6) [47]. Importantly, differential strengths across oxidative/inflammatory markers suggest formulation-specific “efficacy fingerprints.” Among these, Base + U showed the most prominent incremental benefit on lipid peroxidation and inflammatory burden, as evidenced by stronger suppression of MDA and TNF-α (Figure 5), alongside more consistent anti-apoptotic trends at the transcript level (Figure 6). Rather than claiming synergy, which would require component-splitting and interaction testing, these data support an incremental benefit profile for Base + U on selected stress/inflammation axes. Given that Base + U differs from Base by the addition of UA, UA emerges as a plausible contributor to the enhanced stress-containment phenotype. UA is a diet-derived gut microbial metabolite generated from ellagitannins and ellagic acid and has been associated with antioxidant/anti-inflammatory activity, mitochondrial health, and cellular stress regulation in previous studies [14,15,16,48]. In the present study, Base + U showed an incremental benefit on selected oxidative-inflammatory endpoints, particularly MDA and TNF-α, providing a plausible biochemical rationale for the enhanced effect of the UA-enriched formulation. However, mitochondrial function and mitophagy flux were not directly measured here. Therefore, whether mitochondrial quality-control pathways contribute to the observed effects remains a testable hypothesis that should be validated using direct mitochondrial and GC-specific pathway assays.

Several limitations define clear next steps to strengthen causality. First, longer time-course studies are needed to determine whether endocrine restoration and stress containment ultimately translate into more pronounced structural recovery of the follicle pool and more robust central axis remodeling. Second, given that apoptosis reduction was trend-level across some comparisons (Figure 3D), increased statistical power and follicle-stage–matched quantification of GC apoptosis would strengthen mechanistic inference. Third, to test the proposed stress-tolerance mechanism, particularly for Base + U, direct assays of ovarian mitochondrial function (ROS burden, membrane potential, respiration/ATP), mitophagy flux, and steroidogenic enzyme activity in isolated follicles/GCs are warranted. Fourth, only one dose level and one dosing frequency were tested for each intervention in the present study. Therefore, whether higher doses, alternative component ratios, or more frequent administration could produce stronger ovarian protection remains unknown. Future dose-escalation and time-course studies are needed to define the relationship and optimal intervention window for Base and Base + U.

In conclusion, the present data support a mechanistic model in which CTX establishes an oxidative-inflammatory ovarian microenvironment that destabilizes GC survival and follicular fate, impairs steroidogenic competence, and disrupts endocrine feedback. CoQ10, Base, and Base + U partially alleviated this cascade, with coordinated changes in hormone profiles, cyclicity, and ovarian oxidative-inflammatory markers providing supportive evidence of ovarian protection. Notably, Base + U showed an incremental benefit on selected oxidative/inflammatory endpoints, highlighting UA as a potentially important functional component for enhancing ovarian stress resilience under chemotoxic challenge. However, whether these improvements translate into restored fertility or long-term reproductive function remains to be determined.

## Data Availability

The data presented in this study are available on request from the corresponding author. The data are not publicly available due to institutional data-management restrictions and ongoing related research.

## Supplementary Materials

The following supporting information can be downloaded at: https://www.mdpi.com/article/10.3390/antiox15060662/s1, Figure S1: Schematic diagram of the experimental design; Table S1: The primer sequences for qRT-PCR.
