# Supplementary material for: Urolithin A-Enhanced Multi-Bioactive Formulation Mitigates Cyclophosphamide-Induced Premature Ovarian Failure Through Suppression of Oxidative-Inflammatory Stress and Preservation of Follicle Fate
Source: Antioxidants (Basel). 2026 May 24;15(6):662. doi: 10.3390/antiox15060662 (PMC13295883; doi:10.3390/antiox15060662)
Supplement: Supplementary file 1 [file antioxidants-15-00662-s001.zip › antioxidants-4274435-supplementary.pdf]

Supplementary files

Table S1The primer sequences for qRT-PCR

Gene name	Forward (5'-3')	Reverse (5'-3')
β-actin	CTGCTATGTTGCTCTAGACTTCG	ATGCCACAGGATTCCATACC
Bax	CTGATGGCAACTTCAACTG	ATCTTCTTCCAGATGGTGAG
Bcl2	TGGATGACTGAGTACCTGAACC	ATCAAACAGAGGTCGCATGC
Caspase3	GCTGACTTCCTGTATGCTTA	GTTGCCACCTTCCTGTTAA
Gnrh	CATTCTACTGCTGACTGTGT	CCTGGCTTCCTCTTCAATC
Mapk1 (ERK)	CCAGCATTCAGAAGTCAGA	GGATACAGAGGCAAGAAGG
Hsd17b1	CTGTGTTGGATGTGAATGTG	CTTCGTGGAATGGCAGTC
Hsd17b2	CCTGCTTCCTTCTTCTGTAT	TATCTGCTCTGGCTTGGT
FSHR	TATGGCCCTCCTCGTACTCA	AAATTGGGGCCATGCAGAGA
StAR	TCCTCGCTACGTTCAAGCTG	ACGTCGAACTTGACCCATCC
Esr1	TGCTCCTAACTTGCTCCT	GATGTGGTCCTTCTCTTCC
Esr2	CAACCTCCTGATGCTTCTT	TGCTCTTACTGTCCTCTGT
Cyp19a1	CTGAACATCGGAAGAATGC	TCACCTGGAATCGTCTCA

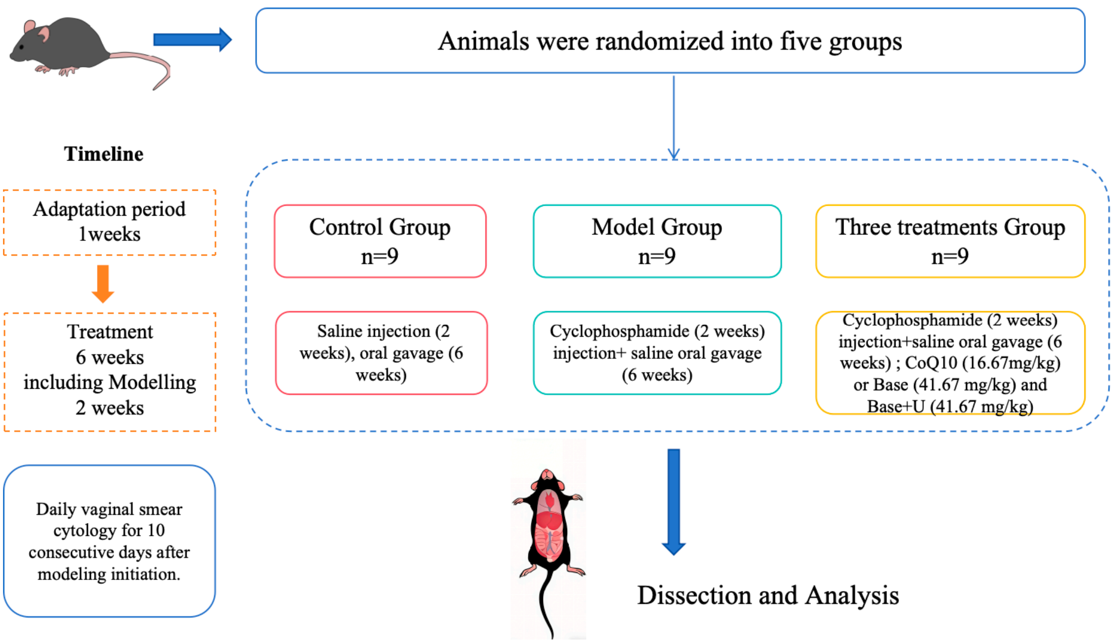

Figure S1: Schematic diagram of the experimental design
